# An Unfinished Chapter: The Impact of Belgians’ Social Representations of Colonialism on their Present-Day Attitudes Towards Congolese People Living in Belgium

**DOI:** 10.5334/irsp.777

**Published:** 2023-08-08

**Authors:** Simona Lastrego, Zoé Magonet, Laurent Licata

**Affiliations:** 1 Université libre de Bruxelles, Belgium (ULB), Belgium

**Keywords:** Collective Memory, Social Representation of Colonialism, Colonialism, Intergroup attitude, Racism

## Abstract

Various national and international political associations have claimed that present-day racism towards people of African descent living in formerly colonizing European countries is caused by these countries’ colonial past and their governments’ failure to adequately address this historical legacy. However, no empirical study has yet examined the relation between social representations of colonialism (SRC) and attitudes towards Afro-descendants among majority group members. To investigate this phenomenon, we carried out two cross-sectional studies, which both confirmed that SRC are associated with attitudes towards the Congolese and with support for compensations. Study 1 (*N* = 407) investigated, among Belgian participants, the association of the two dimensions of SRC – Exploitation and Development – with present-day intergroup attitudes and support for compensations (material and symbolic) for colonialism. Representing the colonial past in terms of Exploitation was associated with more positive attitudes and more intentions to compensate for colonialism, whereas representing it in terms of Development had the opposite effect. Some of these effects were moderated by national identification. Study 2 (*N* = 203) used the same design but also included a modern racism scale. Results of Study 1 were generally replicated, and effects of SRC on modern racism were obtained: SRC in terms of Exploitation was associated with less modern racism, and SRC in terms of Development was associated with more modern racism. Results are discussed in terms of post-colonial intergroup relations.

‘Racism sums up and symbolizes the fundamental relationship that unites the colonialist and the colonized’

Albert Memmi (1957)

As Memmi’s quote underlines, colonialism is fundamentally associated with racism. Indeed, this association was denounced by anti-colonial intellectuals such as Fanon (1952), Césaire (1989), and Memmi (1957) during colonial times. But racism inspired by colonialism did not end with formal colonialism. Indeed, contemporary authors such as Mbembe (2017) or Mignolo (2008), have pointed out the connection between present-day racism and the colonial legacy. This association is also regularly evoked in the political sphere, such as the parliamentary commission on Belgium’s colonial past (Belga, 2022a). For instance, in 2019, a European Parliament resolution asked EU member countries’ institutions to ‘officially acknowledge and mark the histories of people of African descent’ in Europe to combat racism.^1^ A UN Working Group of Experts on People of African Descent was sent in 2019 to Belgium to assess the country’s progress in its fight against racism. The visit ended with a press conference in which the experts stated that ‘The root causes of present-day human rights violations lie in the lack of recognition of the true scope of the violence and injustice of colonization.’^2^ Despite this line of thought, at the end of a two-year examination of its colonial history and its enduring consequences, the Belgian Parliament failed to offer public apologies or compensations for the country’s colonial past, due to ideological disagreements within the Parliament (Belga, 2022b).

One of the various ways in which the colonial past still weighs on contemporary intergroup relations is through its enduring social psychological effects. Indeed, research in social psychology (Licata & Klein, 2005, 2010; see also Licata et al., 2018) have stressed that social representations of colonialism (SRC) are a key element to understand how colonialism is remembered and how it affects present-day intergroup attitudes. However, so far, the association between SRC and present-day attitudes towards immigrants from formerly colonized countries has not been clearly established. Thus, the aim of the present article is to empirically test the assumptions suggesting that the way the colonial past is represented is partly responsible for present-day racism. This study also allows us to overcome a gap in the study of the impact of the colonial past. Indeed, social psychological research has often only focused on negative representations of colonialism to evaluate their impact on intention to offer reparations (Allpress et al., 2010; Brown et al., 2008). Although we will investigate this association, we will also assess the impact of positive representations of colonialism on present-day attitudes, and more specifically their association with negative attitudes, racism and intention to offer reparations.

## Belgium’s Colonial Past in Congo and Afro-Descendants’ Present-Day Situation in Belgium

Officially, the Congo was Belgium’s colony from 1908 to 1960. However, King Leopold II gained control over the territory earlier. During the 1885 Conference of Berlin, the King successfully convinced other European powers to let him extend his control over the Congo under the pretense of humanitarian work (Ndaywel è Nziem, 1998), leading him to become the near private owner of the Congo Free State (Goddeeris et al., 2020). Historians have characterized this period as extremely violent and cruel towards the Congolese population (Stanard, 2019; Burroughs, 2018; Vellut, 2005). At the beginning of the 20th century, a large international humanitarian campaign targeted the King and how the native populations were treated under his regime, such as severed hands, being held as hostages, and children’s abductions (Hochschild, 1998). Thus, in 1908, Leopold II handed over the Congo Free State to Belgium, which in turn became a legal Belgian colony until 1960. Despite this change in government, colonizers and colonized continued to live in a highly segregated environment (Turner, 2007). It was only in 1960, after much struggle, that Congolese independentist movements obtained independence from Belgium and the Republic of Congo was created under the presidency of Joseph Kasa-Vubu, with Patrice Lumumba as the Prime-minister.

As mentioned earlier, Belgium’s colonial past still attracts a lot of criticism at international level and within Belgian society. In 2020, following the Black Lives Matters protests over the murder of George Floyd, the King of Belgium expressed his ‘deepest regrets’ for the ‘acts of violence’ and ‘suffering’ inflicted in colonial Congo. This was the first official expression of such regret.

For a long time, the Congolese immigration remained very limited in Belgium. It was only in the 1990’s that more Congolese people started to immigrate (Mazzocchetti, 2014) and, today, Belgium counts around 250,000 Afro-descendants, with people of Congolese origin representing about half of this community (Demart et al., 2017). In 2017, a study carried out by different Belgian universities showed that, although 60% of Afro-descendants have a higher-level diploma, their level of unemployment is four times higher than that of native Belgians (Demart et al., 2017). The study highlights different types of discriminations that members of this community have endured, ranging from racist comments to denial of basic rights such as housing and working, as well as police brutality. These claims were supported by a study carried out in 2019 by Brussels’s public employment service (Actiris, 2019), which showed that structural mechanisms continuously discriminate the Afro-descendant community, not allowing its members to benefit from the same chances as other communities in terms of work opportunities in Brussels.

Although this article focuses on prejudice towards Afro-descendants, we do not contend that this community is the only one to be discriminated in Belgium, as rightly claimed by Fadil and Martinello (2020). Indeed, colonialism is not the only factor which may explain present-day racism (Stephan & Stephan, 2000). However, since the link between the colonial past and present-day racism has often been suggested, we intend to examine this specific relation, which ties the Congolese people to Belgians.

## Collective Memory and Its Functions

How Belgians *represent* the colonial past, rather than historical accuracy, is a key element in our theoretical reasoning. Collective memory has indeed been defined as ‘a shared set of representations of the past based on a common identity to a group’ (Licata & Klein, 2005: 243) and it allows to underpin the affective, cognitive and behavioral aspects of memory (Figueiredo et al., 2017:.695). Social psychologists often refer to these memories as social representations of history (Liu & Hilton, 2005). Collective memory is often distorted to flatter the ingroup (Baumeister & Hastings, 1997). Indeed, it fulfils three main social psychological functions (Licata & Klein, 2005). Firstly, collective memory gives a sense of identity to a group, answering the question ‘who are we and where do we come from?’ Secondly, it allows to confer and maintain a positive identity, by selecting and presenting flattering aspects of the past (Baumeister & Hastings, 1997; but see Lienen & Cohrs, 2021 for an exception). As a matter of fact, being perceived as a moral person or group is fundamental to individuals, much more than other traits (Brambilla & Leach, 2014). Thus, immoral acts are often denied, distorted and/or justified to maintain the illusion that the ingroup is moral. Thirdly, it is used to justify present-day actions (e.g., war declarations or present-day discriminations can be supported by mobilizing past victimhood).

Although social representations of history are based on ingroup membership, they do not necessarily imply a shared and unanimous version of the past among ingroup members (Lastrego et al., 2022; Kus et al., 2013; Moscovici, 1984). Discordances over collective memory can be sometimes explained by a generational effect (Schuman & Scott, 1989; Licata & Klein, 2010), social class differences (Gaskell & Wright, 1997), different levels of national identification (Licata & Klein, 2010), or even different attitudes towards the historical episode (Lastrego et al., 2022). Discordant collective memories are often observed for colonial collective memories (Licata et al., 2018). For instance, Licata and Klein (2010) found discrepancies over how the colonial past is perceived by young participants, their parents, and their grandparents. Social psychological studies of collective memory mostly focused on unflattering collective memories as they may lead to experiencing collective guilt (Branscombe & Doosje, 2004). In the attempt of reducing such unpleasant emotions, individuals may be motivated to ‘repair’ the ingroup’s harm or to deploy strategies to avoid them (Doosje et al., 1998; Branscombe & Doosje, 2004). However, much less attention has been paid to the impact of positive representations of colonialism in terms of intergroup relations. In our studies, we intend to study how positive and negative social representations of colonialism interact with present-day intergroup relations.

## Social Representations of Colonialism and Their Intergroup Implications

Colonialism can be described as a ‘long traumatic relationship having tremendous influence on the psychologies of both the colonized and the colonizers, deeply affecting their views of the world, of the other peoples, and of themselves’ (Licata, 2012: 1). Denigrating native populations’ culture was a common practice of colonial powers. It allowed colonizers to consider that only their culture had value and to despise the culture of the indigenous peoples (Fanon, 1952; Said, 1993). Colonial propaganda played an important role in spreading prejudice (M’Bokolo & Truddaïu, 2018), thus allowing the justification of colonial missions. However, although many would agree with this definition, others would vehemently disagree since the colonial past is subject to strong controversies and embedded into strong ideological standpoints (Rosoux & van Ypersele, 2011; Lastrego et al., 2022). Until the 1990’s, Belgians had generally a rather positive image of their colonial past, but after 1998, marked by the publication of Hochschild’s bestseller *King Leopold’s Ghost*, which narrated Belgians’ actions in Congo in terms of genocide, Belgians started to hold a wider range of opinions on what happened during their colonial past (Licata & Klein, 2010).

According to Licata and Klein (2010), social representations of colonialism are structured around two dimensions: one of ‘Development’ in the fields of economy, education, or health care, and one of ‘Exploitation’ of the human lives and natural resources of the colonized countries, which also encompasses racial segregation, massacres, and atrocities. The bi-dimensional feature of SRC was found among different European – including Belgians – as well as African samples (Licata et al., 2018). In the European samples, SRC in terms of Exploitation positively predicted guilt and shame for colonialism, as well as support for reparation, whereas SRC in terms of Development had the opposite effect. However, this study did not address attitudes towards the formerly colonized people.

The relation between colonial collective memory and present-day intergroup attitudes has been suggested by various social psychologists (Brasil & Cabecinhas; 2018; Licata, 2012; Volpato & Licata, 2010; Figueiredo et al., 2013). However, the only ones who came closer to empirical evidence are Vala et al. (2008), who linked adherence to luso-tropicalism and present-day prejudice. Luso-tropicalism is a myth portraying Portuguese colonizers as having been particularly friendly to colonized people (Valentim & Heleno, 2018). These authors found an association between adherence to luso-tropicalist ideology and the cultural inferiorization of Black African immigrants (thus prejudice against this community), suggesting that the way colonialism is perceived has an impact on present intergroup relations. Although it does not address SRC, research conducted in the US about the Marley hypothesis (Bonam et al., 2019; Nelson et al., 2013) is also relevant. This hypothesis, based on a song from the famous Jamaican singer, states that ignorance of historical racist events is responsible for White people’s failure to perceive today’s enduring racism. Accordingly, these studies showed that Black participants remembered more historical racist events than White ones, and that this difference mediated the effect of racialized group belonging on perception of systemic manifestations of racism (Nelson et al., 2013). Thus, the lack of critical historical consciousness was associated with denial of racism. To sum up, research on luso-tropicalism showed that positive representations of the colonial past can be instrumental in legitimizing colonialism, which fuels prejudice towards African immigrants. Conversely, research on the Marley hypothesis shows that critical historical knowledge may have the opposite effect. Applied to social representations of Belgium’s colonial past, we expect that SRC in terms of Development – i.e., a positive representation of the colonial action in Congo – will be associated with more negative attitudes and with more racism towards Congolese immigrants, whereas SRC in terms of Exploitation – i.e., a critical representation of Belgian colonialism – will be associated with more positive attitudes and less racism.

## Overview

After the UN Working Group visit, Belgium’s government decided to reflect on how Belgium’s colonial past influences present-day relationship towards the Afro-descendant community and promised to look at the impact of colonialism on structural discrimination. Indeed, in 2020 the parliament mandated its own group of experts to investigate the long-term consequences of colonialism. Our goal is to feed this reflection by investigating the relationship between SRC and present-day attitudes towards people or Congolese origin living in Belgium. By doing so, we want to empirically test the link between SRC and present-day racism, thus adopting a micro-level perspective to analyze such phenomenon. To do so, we carried out two cross-sectional studies in which we measured SRC and present-day attitudes towards the Congolese of Belgium (Study 1) and modern racism (Study 2). In both studies, we used the Social Representation of Colonialism Scale (Licata et al., 2018), which presents two dimensions of colonialism: One focusing on the positive aspects pertaining to the Development (civilizing missions, development of infrastructures, education, and health services) of colonized countries and the other on the negative aspects pertaining to the Exploitation (racism, exploitation of the natural resources and the workforce, physical and moral abuses). We contend that present attitude towards the Congolese should vary depending on the focus of participants’ colonial collective memory (development versus exploitation).

## Study 1

For this first study, we aimed to recruit participants holding different attitudes towards colonialism. To do so, we recruited University participants, who generally hold critical memories, focusing on the exploitation aspects (Licata & Klein, 2010) and participants who belong to a pro-colonial association (Mémoires du Congo, du Rwanda et du Burundi), who should score higher on the developmental aspects of colonial collective memory.

In this study, we contend that SRC (Development and Exploitation) is associated with Belgian participants’ present-day intergroup attitudes towards the Congolese community of Belgium (H1). We expect (H1a) higher scores of SRC Development to predict negative present-day attitudes towards the Congolese community, whereas we expect higher scores of SRC Exploitation to predict positive present-day attitudes (H1b). We then expect (H2) SRC to impact Belgians’ intention to compensate Congolese people, through excuses and material reparations, as observed in previous research (Licata et al., 2018). Thus, we expect (H2a) higher scores of SRC Development to predict lower intention to compensate, whereas we expect higher scores of SRC Exploitation to predict greater intention to compensate (H2b). Finally, (H3) in accordance with findings from Licata & Klein (2010), we expect results tested by H1 and H2 to be moderated by Belgian Identification. Individuals who identify more with their country also need more to protect themselves from negative aspects of their country’s colonial actions compared to lower identifiers. Thus, the effects of SRC Development should be higher for high identifiers, whereas the effects of SRC Exploitation should be lower for high identifiers than for low identifiers.

### Participants

Our sample originally included 414 Belgian participants. However, we removed seven participants with Congolese origins. Our final sample was thus composed of 407 participants (312 women and 95 men). The average age is 28.4 years (*SD* = 19.70). However, the sample’s age varies greatly: From the youngest being 18 to the oldest being 91. Participants were recruited at university (*N* = 345) and among colonial associations (*N* = 62). Among our participants, 55 stated that they had lived in Africa, while 192 indicated that they had friends and/or relatives that have lived in Africa. The sample size of Study 1 allows to detect a minimum correlation of *r* = .16, with a power of .90 and two-tailed tests.

### Measures and Procedure

We recruited first year psychology students of the Université libre de Bruxelles by inviting them to participate in an online survey on the history of Belgian colonialism in Congo. In exchange, students received course credits (withdrawal from the study was possible at any given time without compromising the credit’s allocation). At the same time, we shared the link for our questionnaire with the president of a pro-colonial association (Mémoires du Congo du Rwanda et du Burundi), whose members are former settlers or members of their family. The president forwarded the study’s link through the association newsletter, asking members of the association to complete the questionnaire for educational and research purposes. This was done in order to recruit participants who presumably would hold more positive attitudes towards colonialism than students (Licata & Klein, 2010). However, this entailed using different recruitment procedures, which we acknowledge as a limitation.

All the items used in both studies are available in the Supplementary Materials. Except when indicated, all items were rated on a seven-point scale (1 = strongly disagree, 7 = strongly agree). Participants were first asked to indicate their general attitude towards colonialism. *Attitude towards colonialism* was measured by asking: ‘Overall, what is your attitude towards colonialism?’ Participants answered by choosing between two options: ‘favorable’ or ‘unfavorable.’ This measure was included to make sure that different attitudes towards colonialism were represented in the sample. It will not be used in subsequent analyses.

*Social representation of colonialism (SRC)*: was adapted from Licata et al. scale (2018). Participants were asked ‘When you think about the Belgian colonization of the Congo, how strongly do you agree with each statement?.’ As suggested by Licata and Klein (2010), SRC presents two dimensions: Negative aspects of colonialism, such as abuse and violence (SRC Exploitation) and positive aspects related to development (SRC Development). Both SRC Exploitation (*α* = .88; e.g., ‘Exploitation of Congo’s resources for Belgium’s benefit’) and SRC Development (*α* = .70; e.g., ‘Construction of health and school systems’) were composed of five items.

*Intention to compensate* was adapted from Allpress et al. (2010) and presented two dimensions: *Material compensation* (three items, *α* = .90; e.g. ‘I am in favor of the Belgian government offering financial compensation to the Congolese for past injustices’) and *Excuses* (two items, *α* = .87; e.g. ‘Belgian government should publicly apologize for the misdeeds committed during its colonial past’). Both dimensions measure participants’ support for the Belgian government to present material and moral compensations, not intention to offer personal compensations.

*Attitude towards Congolese community* is an affective measure of intergroup attitude inspired by Abelson et al. (1982). Participants were presented with a metaphorical thermometer, which has the ability to measure their attitudinal temperature towards the members of the Congolese community of Belgium: ‘Imagine you could translate your general attitude towards the members of the Congolese community of Belgium under the form of a temperature. This can be very warm (100° = extremely favorable) or very cold (0° = extremely unfavorable). Please indicate the degree that best expresses your general attitude towards the Congolese community of Belgium.’

*Belgian Identification* was measured with a four-item scale (e.g., ‘I identify to Belgium’) (α = .87) adapted from Brown et al. (1986).

Finally, participants were asked to indicate their age, gender, and nationality.

### Results

#### Descriptives

Supplementary Materials include Table 1 with means and standard deviations for all dependent variables according to participants’ attitude towards colonialism and Table 2 with correlations for all measured variables.

Seventy-five participants were in favor and 332 in disfavor of colonialism, indicating a reasonable variety of views.

#### Testing of Hypotheses

To test our first hypothesis, we carried out a multiple regression analysis, predicting attitude towards the Congolese based on both components of colonial collective memory: SRC Development and Exploitation. The general model was significant: *F*(2, 404) = 16.23, *p* < .001, *R^2^* = .07. As expected, the two dimensions of colonial collective memory impact differently present-day attitude towards the Congolese community: (H1a) Higher scores of SRC Exploitation predict a warmer (more positive) attitude towards the Congolese, *b* = 3.48, 95% CI [1.97, 4.99], *t*(404) = 4.53, *p* < .001, *ηp^2^* = .04, whereas (H1b) higher scores of SRC Development predict a colder attitude, *b* = –1.91, 95% CI [–3.64, –0.18], *t*(404) = –2.17, *p* < .05, *ηp^2^* = .01.

We then carried out the same statistical analysis to test H2, analyzing the impact of colonial collective memory on support for compensation. We first looked at support for material reparations; the analysis yielded a significant general model: *F* (2, 404) = 59.04, *p* < .001, *R^2^* = .23. As expected (H2a), higher scores of SRC Exploitation predict higher support for material reparations, *b* = .34, 95% CI [.23, .46], *t*(404) = 5.85, *p* < .001, *ηp^2^* =.07, whereas (H2b) higher scores of SRC Development predict lower support for material reparations, *b* = –.49, 95% CI [–.62, –0.35], t(404) = –7.34, *p* < .001, *ηp^2^* =.11. The same pattern was observed for support for excuses, *F* (2, 404) = 82.98, *p* < .001, *R^2^* = .29, as SRC Exploitation positively predicts a support for excuses, *b* = .54, 95% CI [.43, .65], *t*(404) = 9.58, *p* < .001, *ηp^2^* =.18, while SRC Development negatively predicts it, *b* = –.38, 95% CI [–.51, –.25], *t*(404) = –5.85, *p* < .001, *ηp^2^* =.07.

Finally, we tested for the moderating effect of National identification (H3). The general model predicting Attitude towards the Congolese was significant, *F* (5, 402) = 6.61, *p* < .001, *R^2^* = .08, but neither the effects of national identification nor of its interaction with SRC Development and SRC Exploitation were significant. Table 3 presents all coefficients of the moderation analyses. Only SRC Exploitation had a significant independent effect on Attitude towards the Congolese, while the effect of SRC Development was only marginally significant (*p* = .06).

**Table 3:** Moderation analysis for Study 1.


	ATTITUDE TOWARDS THE CONGOLESE			MATERIAL COMPENSATION			SYMBOLIC COMPENSATION (EXCUSES)		
		
	*b*	t	95%CI	*b*	t	95%CI	*b*	t	95%CI

SRC Development	–1.78	–1.87	[–3.65, .09]	–.46	–6.46***	[–.60, –.32]	–.32	–4.70***	[–.45, –.18]

SRC Exploitation	3.53	4.54***	[2.0, .5.05]	.33	5.60***	[.21, .44]	.52	9.32***	[.41, .63]

National identification	–.14	–.18	[–1.67, 1.39]	–.03	–.49	[–.14, .08]	–.08	–1.46	[–.19, .02]

Development* identification	.35	.58	[–.84, 1.53]	–.11	–2.45*	[–.19, –.02]	–.11	–2.60**	[–.19, –.02]

Exploitation* identification	.44	.79	[–.65, .1.53]	.05	1.30	[–.02, .13]	.07	1.81	[–.01, .15]


*Note*: * *p* < .05, ** *p* < .01, *** *p* < .001.

We then carried out the same moderation analysis with material compensation as dependent variable; the general model was significant, *F*(5,402) = 26.52, *p* < .001, *R^2^* = .25. As Table 3 shows, both SRC Development and Exploitation had significant effects. National identification had no significant effect, but the interaction between National identification and SRC Development was significant. To analyze the simple effects of such interaction, we carried a moderation analysis via PROCESS (Hayes, 2017, model 1). SRC Development was the independent variable, material compensation was the dependent one, National identification was entered as the moderator and SRC Exploitation was entered as covariate. The analysis indicated that the impact of SRC Development on predicting a lower intention to offer compensation was significant for both high, *b* = –.67, 95% CI [–.94, –.60], *t*(404) = –7.38, *p* < .001 and low identifiers, *b* = –.26, 95% CI [–.51, –.15], *t* (404) = –2.66, *p* < .01, although the effect was stronger for high identifiers.

Then, another moderation analysis was carried out for excuses, *F*(5,402) = 37.77, *p* < .001, *R^2^*=.32. We obtained the same pattern of results as for Material compensations. Once again, we carried a moderation analysis via PROCESS (Hayes, 2017, model 1) to analyze the simple effects of the moderation. The analysis indicated that the impact of SRC Development was significant on predicting a low intention to offer excuses only for high identifiers, *b* = –.54, 95% CI [–.90, –.55], *t*(404) = –6.27, *p* < .001, but not for low identifiers, *b* = –.11, 95% CI [–.40, –.03], *t*(404) = –1.18, *p* =.24.

### Discussion of Study 1

Study 1 allowed to confirm most of the tested hypotheses. Indeed, participants who focus on the developmental aspects of SRC expressed a colder attitude towards members of the present-day Congolese community and expressed lower support for compensation, both in terms of excuses and material reparations. In contrast, those who focus on exploitation expressed a warmer attitude towards them, as well as higher support for offering them material or symbolic forms of compensation. These results are thus compatible with the idea that how the colonial past is represented, plays an important role in present intergroup relations, as suggested by the European Parliament resolution and the UN Working Group of Experts on People of African Descent. We also expected to observe these effects to be moderated by Belgian Identification, as different studies have shown an association between colonial collective memory and national identification (Licata & Klein, 2010; Licata et al., 2018). However, this moderation effect was observed only on the impact of SRC Development on support for material and for symbolic compensation. Hence, the negative effect of SRC Development on support for compensation was stronger among high national identifiers. However, contrary to hypothesis, National identification did not moderate the effect of SRC exploitation on support for material or symbolic compensation.

Even though results confirmed most hypotheses, Study1 presents two main limitations. Firstly, our sample was not sufficiently representative as it was composed of two specific sub-samples (i.e., university students and members of a pro-colonial association). Secondly, the study did not include a proper measure of racism, focusing on intergroup attitude instead. Thus, we carried a second study to overcome these issues.

## Study 2

This second study’s goal was to clarify the association between SRC and present-day racism, in addition to intergroup attitude, as tested in Study 1. In modern Western societies, norms of respect and equality tend to be encouraged, and expressions of blatant racism are judged negatively, if not legally prohibited. However, racism is still widespread, even though it tends to be expressed in more subtle ways (Pettigrew & Meertens, 1995; Pearson et al., 2009). Indeed, while many White individuals support principles of racial equality and consider themselves as non-prejudiced, they still have, although often non-consciously, negative feelings and beliefs about Black people (Gaertner & Dovidio, 1986; Pearson et al., 2009). Thus, in Study 2, we added a modern-racism scale (McConahay, 1986). However, we did not include the measures of support for symbolic and material compensation. In addition, we recruited a non-student sample.

In this second study, similarly to Study 1, we expected that: (H1a) SRC Development will be associated with colder attitudes towards members of the Congolese community whereas (H1b) SRC Exploitation will have the opposite effect. We also expected that: (H2a) SRC Development will predict higher scores of modern racism whereas (H2b) SRC Exploitation will have the opposite effect. As in Study 1, we will also test (H3) for a moderation effect of National identification on the effects tested for testing H1 and H2.

### Participants

Our sample originally included 210 Belgian participants, but we removed seven individuals with Congolese origins. Our final sample thus comprises 203 participants (142 women, 60 men, and 1 non-binary person). Participants belonged to different age groups ranging from 18 to over 55: The majority of them belonged to the age group of ‘45–54 years old’ (*N* = 79), followed by ‘55 and over’ (*N* = 54), then by ‘25–34 years old’ (*N* = 35) and finally the last two age groups ‘35–44 years old’ (*N* = 18) and ‘18–24 years old’ (*N* = 17). The sample was composed of 118 employees, 28 self-employed, 24 retired individuals, 17 students, 3 workmen, and 13 individuals that did not share their professional status. Only 11 had lived in the Congo, whereas 96 indicated that they had friends and/or relatives that had lived in the Congo. The sample size of Study 2 allows to detect a minimum correlation of *r* =.23, with a power of .90 and two-tailed tests.

### Measures and Procedure

The questionnaire was available online and its weblink was mainly shared through one of the authors’ social network profile, but also by email among her contacts in order to reach people with no access to social networks. Participants were informed, as in Study 1, that the study investigated their opinion on Belgian colonialism in the Congo.

Measures of Belgian identification, SRC (Development and Exploitation) as well as Attitude towards members of the Congolese community were the same as the ones used in Study 1. The only difference is that Study 2 did not include measures of support for compensation but included a scale of *Modern Racism* adapted from McConahay (1986) and designed to estimate the cognitive dimension of racist attitudes. This 10-item scale (*α* = .84) was used to evaluate Belgians’ modern racism towards members of the Congolese diaspora; some items were reversed, meaning that high scores for those specific items indicate higher racism (e.g., ‘Today, there is no more discrimination against the Congolese community in Belgium’).

### Results

#### Descriptives

SRC Exploitation scores, *M* = 5.13, *SD* = 1.24, *t*(202) = 58.9, *p* < .001, *d* = 4.13, suggest that our sample tends to agree with the more negative aspect of social representations of colonialism. In comparison, on average, participants neither agreed nor disagreed with SRC Development, *M* = 4.17, *SD* = 1.14, *t*(202) = 52.1, *p* < .001, *d* = 3.66. This is also the case for modern racism, *M* = 3.74, *SD* = 0.86, *t*(202) = 61.7, *p* < .001, *d* = 4.33. For identification with Belgium, respondents seem to generally identify with Belgium, *M* = 5.49, *SD* = 0.82 *t*(202) = 95.6, *p* < .001, *d* = 6.71. Finally, the temperature of attitudes towards the Congolese diaspora in Belgium indicates a moderately average score, *M* = 62.21, *SD* = 31.55 *t*(202) = 28.1, *p* < .001, *d* = 41.97, moderately positive. Supplementary Materials include Table 4, which presents correlations between all measured variables.

#### Testing of Hypotheses

To test our first hypotheses (H1a and b), we carried out a series of multiple regression analyses. The model explored the effect of both SRC Development and Exploitation on attitude towards the Congolese, *F* (2, 200) = 10.54, *p* < .001, *R^2^* = .10. As expected, the more participants held SRC in terms of exploitation, the warmer were their attitudes towards the Congolese, *b* = 4, 95% CI [3.69, 10.87], *t*(200) = 7.29, *p* < .001, *ηp^2^* =.07. However, contrary to our hypothesis, SRC Development did not significantly predict colder attitudes, *b* = –1.71, 95% CI [–5.6, 2.19], *t*(200) = –.86, *p* = .39, *ηp^2^* =.05.

Then, we carried out the same analysis with modern racism as the dependent variable (H2a and b): *F* (2, 200) = 48.91, *p* < .001, *R^2^* = .33. As expected, the more participants held a SRC in terms of exploitation, the less they scored on the modern racism scale, *b* = –.31, 95% CI [–.38, –.21], *t* (202) = –7.33, *p* < .001, *ηp^2^* =.19. In contrast, the more they held SRC in terms of development, the higher they scored on the modern racism scale, *b* = .17, 95% CI [.11, .29], *t*(200) = 3.63, *p* < .001, *ηp^2^* =.08.

Finally, we tested the moderation effect of National identification on the effects tested in H1 and H2, as shown in Table 5. We first carried out a multiple regression analysis with attitude towards the Congolese community as the dependent variable: *F* (5,197) = 4.68, *p* < .001, *R^2^* = .11. As Table 4 shows, only SRC Exploitation remained significant. Thus, National identification had no independent effect on present-day attitude towards the Congolese and did not moderate the effect of the SRC.

**Table 5:** Moderation analysis for Study 2.


	ATTITUDE TOWARDS THE CONGOLESE			MODERN RACISM		
	
	*b*	t	95%CI	*b*	t	95%CI

SRC Development	–1.29	–.62	[–5.37, 2.79]	.19	3.84***	[.09, .28]

SRC Exploitation	7.21	3.94***	[3.60, 10.82]	–.30	–6.85***	[–.38, –.21]

National identification	–2.18	–.79	[–7.64, 3.28]	.09	1.34	[–.04, .21]

Development* identification	–1.62	–.69	[–6.21, 2.98]	.02	.35	[–.08, .12]

Exploitation* identification	–2.08	–.96	[–6.37, 2.21]	.01	.25	[–.08, .11]


*Note*: * *p* < .05, ** *p* < .01, *** *p* < .001.

We then performed the same moderation analysis with modern racism as the dependent variable: *F* (5,197) = 20.35, *p* < .001, *R^2^* = .34. Only SRC Exploitation and Development significantly predicted modern racism, in the expected directions, whereas National identification had no significant independent nor moderating effect.

### Conclusion Study 2

Study 2 partly supported conclusions drawn from Study 1. Indeed, it showed that participants whose SRC focuses on exploitation expressed warmer attitudes towards the Congolese of Belgium and scored lower in terms of modern racism. However, for participants holding SRC focusing on developmental aspects, we observed a significant positive effect on modern racism, but not on attitude towards the Congolese. In addition, and in line with Study 1’s results regarding intergroup attitude, none of these results were moderated by national identification.

## General Discussion

As intellectuals, militants, historians and politicians have often suggested the idea that present-day racism in formerly colonizing nations is linked to the colonial past and its legacies, we decided to investigate the association between social representations of colonial history and present-day intergroup attitudes of Belgian participants towards members of the formerly colonized group currently living in Belgium, the formerly colonizing country. To test such association, we carried out two cross-sectional studies. Study 1 indeed showed that the two dimensions of SRC (Development and Exploitation) have an impact on Belgian participants’ present-day attitudes towards members of the Congolese community, and their support for compensation to the Congolese for the harm inflicted to them during colonialism. More particularly, these findings showed that participants who scored higher on the developmental aspects expressed colder attitudes toward the Congolese and supported symbolic and material compensation to a lesser extent. In contrast, those with higher scores for exploitation expressed warmer present-day attitudes towards the Congolese community and more support for symbolic and material compensation. However, these effects were partly moderated by national identification. Indeed, the negative effect of SRC Development on support for compensation was stronger among high national identifiers, which is consistent with the idea that high identifiers rely more on legitimizing collective memories to avoid having to face their ingroup’s responsibility during colonialism, thus escaping the duty to repair through compensations. However, National identification did not moderate the effects of SRC Exploitation on any of the dependent variables. Thus, holding a critical view on colonial history seems to have positive effects, irrespective of participants’ motivation to protect their national identity. In other words, high national identification tends to amplify the protective effects of the positive dimension of SRC, thus legitimizing their opposition to compensation. In contrast, it does not mitigate the positive effects of a critical version of colonial history on support for compensation.

These results tend to confirm that SRC are linked with levels of prejudice towards Congolese people. However, Study 1 did not include a direct measure of racism and the sample was composed of two specific groups: psychology students and members of an association of former colonials. Thus, we carried out a second study with a more diverse Belgian sample and included a modern racism scale. In addition to replicate the model for attitudes towards the Congolese living in Belgium, Study 2 showed that participants who scored higher on SRC Development also scored higher on modern racism, whereas participants who scored higher in SRC Exploitation scored lower on modern racism. National identification had no significant independent effect on these variables, nor did it significantly interact with the two dimensions of SRC.

Lastrego et al. (2022) have already shown that attitude towards colonialism impacts the narrative structure of this historical chapter. With the present article, we complement this finding by showing present-day repercussion of social representations of colonial history on intergroup relations. As suggested by decolonial activists and scholars, we conclude, based on empirical findings, that the way the colonial past is represented has a significant impact on present-day intergroup relations and that, regarding the effect of the positive representation of colonialism, this is even more the case for high national identifiers (though only in Study 1).

However, the article presents some limitations. First of all, both studies are cross-sectional, casting potential doubts on the causal relation that we hypothesized, as well as on their direction. Indeed, it could be argued that intergroup attitudes and endorsement of racism inspire social representations of colonial history rather than the other way around. To address such issue, experimental studies are needed. Secondly, a great amount of research investigating reparations has indicated the predominant role of collective emotions such as collective guilt (Doosje et al., 1998) and shame (Allpress, et al., 2010). These group-based emotions may mediate the effects reported in the present paper, which could be tested in future studies. In a similar perspective, further studies could integrate variables which are known to be associated to negative intergroup attitudes, such as social dominance orientation, right-wing authoritarianism, or realistic and symbolic threats (Stephan & Stephan, 2000), to ascertain that social representations of colonialism have independent effects over and above these variables. Thirdly, our sample did not present enough participants to compare generations, and data collection was not standardized in study 1. Since the colonial past is highly influenced by participants’ age (Licata & Klein, 2010), further studies using larger and more diversified samples, especially in terms of generations, are needed. In addition, ideally, the order of presentation of the items could be controlled for. Fourthly, to make sure that SRC of colonialism have a specific effect on prejudice towards formerly colonized people, future research should include measures of attitude or racism towards other outgroups. Finally, similar studies should be carried out in other national contexts to check for the generalizability of the phenomenon beyond the Belgian context. Conversely, future studies could investigate how formerly colonized people’s (living in formerly colonized countries or in formerly colonizing ones) collective memories of colonialism also impacts how they perceive the former colonizers and thus obtain a more global perspective on the phenomenon (see Figueiredo et al., 2018; Lastrego, et al., 2023).

While more research is clearly needed in this area, this is, to our knowledge, the first article to empirically investigate the impact of social representations of colonialism on the current attitudes of members of a former colonizing country towards members of a former colonized country – an association that has often been assumed, but still needed empirical evidence. These findings underscore the need to promote a critical historical awareness of colonialism, as well as awareness of its lasting effects on current intergroup relations in multicultural societies (Nelson et al., 2013). Pedagogical methods that convey a critical representation of the colonial past may prove effective. Research has shown that exposure to negative aspects of colonial violence is necessary to foster historical awareness, especially when these episodes are denied (Leone, 2017). Hence, combating racism through history education is far from an easy task, but it is necessary.

## Additional File

The additional file for this article can be found as follows:

10.5334/irsp.777.s1 Supplemental Materials. Tables 1, 2 and 4.

## Footnotes

The resolution’s text is available on: https://www.europarl.europa.eu/doceo/document/TA-8-2019-0239_EN.pdf.

The statement is available on: https://www.ohchr.org/en/statements/2019/02/statement-media-united-nations-working-group-experts-people-african-descent.
