# Supplementary material for: An Unfinished Chapter: The Impact of Belgians’ Social Representations of Colonialism on their Present-Day Attitudes Towards Congolese People Living in Belgium
Source: Int Rev Soc Psychol. 2023 Aug 8;36:13. doi: 10.5334/irsp.777 (PMC12372743; doi:10.5334/irsp.777)
Supplement: Supplemental Materials. — Tables 1, 2 and 4. [file irsp-36-777-s1.pdf]

Supplementary Materials

Table 1

Means and standard for Study 1, according to colonial attitude

Variable	In disfavor of colonialism (*N* = 334)	In favor of colonialism (*N* = 75)	T-test	P value
SRC Development	3.91 (1.06)	5.12 (1.12)	8.82	<.001
SRC Exploitation	5.96 (1.11)	4.30 (1.42)	-11.13	<.001
Material Compensation	5.58 (1.39)	3.34 (1.90)	-11.71	<.001
Excuses	5.97 (1.31)	3.37 (1.85)	-14.24	<.001
Belgian Identification	4.43 (1.31)	4.99 (1.65)	3.18	.002
Attitude towards Congolese	80.48 (19.88)	65.31 (20.39)	-5.94	<.001

Table 2

Correlations for Study 1

Variable	1	2	3	4	5	6	7
1. SRC Development							
2. SRC Exploitation	-.26***						
3. Material compensation	-.40***	.35***					
4. Symbolic compensation	-.36***	.48***	.64***				
5. Belgian Identification	.38***	-.15**	-.17***	-.20***			
6. Attitudes towards the Congolese	-.17***	.25***	.33***	.36***	-.09		
7. Age	.46***	-.31***	-.52***	-.43***	.25***	-.21***	

Table 4

Correlations for Study 2

Variable	1	2	3	4	5
1. SRC Development					
2. SRC Exploitation	-.15*				
3. Modern Racism	.30***	-.54***			
4. Belgian Identification	.29***	-.15*	.20**		
5. Attitude towards the Congolese	-.04	.28**	-.39***	-.14*	
5. Age	.12	-.13	.22**	.09	-.33***

All items used in Study 1 and 2 are available below:

Colonial Attitude was measured by asking : “Overall, what is your attitude towards colonization”. Participants answered by choosing between two options: “favorable” or “unfavorable”.

Social representation of colonialism (SRC): adapted from Licata et al.’s scale (2018). Participants were asked “When you think about the Belgian colonization of the Congo, how strongly do you agree with each statement”:

1. Development of channels of communication and economic infrastructure (SRC Development).
2. Construction of health and school systems. (SRC Development).
3. Exploitation of Congolese labor force by Belgium. (SRC Exploitation).
4. Exploitation of Congo's resources for Belgium’s benefit. (SRC Exploitation).
5. Pacification of the Congo. (SRC Development).
6. Evangelization of the Congo. (SRC Development).
7. Destruction of indigenous Congolese cultures and traditions. (SRC Exploitation).
8. Civilizing missions by Belgians. (SRC Development).
9. Racist attitude of the Belgian colonizers towards the Congolese. (SRC Exploitation).
10. Bad treatment inflicted on the Congolese by the Belgians. (SRC Exploitation).

Intention to compensate: adapted from Allpress et al. (2010).

1. I am in favor of the Belgian government offering financial compensation to the Congolese for past injustices.
2. I am in favor of the Belgian government offering material reparations to the Congolese.
3. The Belgian government should not offer financial or material reparations to the Congolese.
4. The Belgian government should publicly apologize for the misdeeds committed during colonization.
5. There is no reason for the Belgian government to publicly apologize to the Congo about colonization.

Attitude towards Congolese community : is an affective measure of intergroup attitude inspired by Abelson et al. (1982). Participants are presented with a metaphorical thermometer, which has the ability to measure their attitudinal temperature towards the Congolese community of Belgium : “Imagine you could translate your general attitude towards the members of the Congolese community of Belgium under the form of a temperature. This can be very warm (100° = extremely favorable) or very cold (0° = extremely unfavorable). Please

indicate the degree that best expresses your general attitude towards the Congolese community of Belgium."

Belgian Identification was measured with a four-item scale, adapted from Brown et al. (1986):

1. Being "Belgian" is an important aspect of how I see myself.
2. When someone criticizes Belgians, I take it as a personal insult.
3. I like the idea of picturing myself as "Belgian".
4. I feel attachment for the Belgian people.

Modern Racism: adapted from McConahay (1986):

1. Today, there is no longer any discrimination against the Congolese community in Belgium.
2. It's easy to understand the anger felt by the Congolese community in Belgium.
3. Congolese people living in Belgium do not claim their equal rights enough.
4. The Congolese community in Belgium has the right to impose itself.
5. The Congolese community in Belgium is getting more financial aid than it should.
6. In recent years, the Congolese community has not been respected enough by the media and the government.
7. Currently, the Congolese diaspora living in Belgium is confronted on a daily basis with multiple discriminations in various fields (e.g. employment).
8. Currently, the Congolese community is part of Belgium.
9. Today, people of Congolese origin endure unequal treatment or discrimination in Belgium due to their origin.
10. I trust people who belong to the Congolese diaspora in Belgium.
